# DPP-Mediated Interaction of TAZ/β-Catenin Promotes the Differentiation of DPSCs into Odontoblasts

**DOI:** 10.3390/ijms27104599

**Published:** 2026-05-20

**Authors:** Yinghua Chen, Adrienn Petho, Amudha Ganapathy, Velavan Bakthavachalam, Cassandra Villani, Anne George

**Affiliations:** Department of Oral Biology, University of Illinois Chicago, Chicago, IL 60612, USA

**Keywords:** dental pulp stem cell, odontogenic differentiation, tooth development, regeneration, dentin phosphophoryn, NF-κB signaling cascade, TAZ signaling pathway, Wnt/β-catenin pathway

## Abstract

Dental pulp tissue contains mesenchymal stem/progenitor cells that possess high proliferative potential for self-renewal. They are neural-crest derived cells and exhibit multi-lineage differentiation properties. These progenitor stem cells are now recognized as being vital to the dentin regeneration process following injury. Understanding the molecular mechanisms that mediate the differentiation of adult stem cells into odontoblasts and their use in the repair of the dentin–pulp complex is of significant interest in regenerative dental medicine. Dentin Phosphophoryn (DPP), synthesized and processed predominantly by the odontoblasts, functions both as a structural and signaling protein. We had previously demonstrated that DPP activates NF-κB and promotes Wnt5a expression in dental pulp stem cells. In this context, we observed that DPP can activate TAZ, a biologically potent transcriptional coactivator which serves as a downstream element of the NF-κB signaling cascade. Furthermore, binding of NF-κB p65 subunit to the TAZ promoter was facilitated by DPP stimulation, and their interaction was confirmed by ChIP analysis. In addition, DPP-dependent activation of the TAZ/TEAD reporter was confirmed by luciferase activity in DPSCs. Co-immunoprecipitation analysis confirmed the in vivo interaction between TAZ and β-catenin with DPP stimulation. This regulatory complex facilitated TAZ to bind to the conserved TEAD binding motifs of key gene targets involved in odontogenic differentiation such as *RUNX2*, *OSX*, *OCN*, *ALP*, *BMP4*, and WNT5A. Some of these genes also contain binding sites for the TCF/LEF transcription factors that interact with the Wnt effector, β-catenin. Activation of TAZ and β-catenin resulted in the upregulation of odontoblast gene expression and reduced expression in the presence of the TAZ–TEAD protein complex inhibitor. Using mandibles of DSPP KO and WT mice, we confirmed reduced TAZ and β-catenin protein levels in the dental pulp cells and in the odontoblasts of DSPP KO mice when compared with WT. Thus, DPP, an extracellular matrix protein, provides biological cues to activate the TAZ signaling pathway that can stimulate the terminal differentiation of DPSCs into functional odontoblasts.

## 1. Introduction

During the development of the dentin–pulp complex, the ectomesenchymal cells give rise to the dentin, pulp, and periodontal tissue [1]. The dental pulp is a connective tissue and is encased by mineralized dentin [2]. Dentin acts as a barrier by preventing the entry of oral bacteria and viruses into the pulp space. Dentin, a calcified connective tissue, consists of 70% inorganic hydroxyapatite, and 30% of organic matrix and water [3,4]. Noncollagenous proteins form an important functional component of the organic matrix [5,6]. The dental pulp stem cells (DPSCs) confined in the dental pulp are highly proliferative, self-renewable, and possess multi-lineage differentiation potential [7,8,9]. Understanding the molecular mechanism that mediates the differentiation of adult stem cells into odontoblasts and their tissue’s regenerative property makes it a good source for repair and regeneration of the dentin–pulp complex which is of vital interest in regenerative dental medicine [10].

Dentin Phosphophoryn (DPP), an extracellular matrix protein, synthesized predominantly by the odontoblasts, has been the archetype of macromolecules that regulate the biomineralization process [11,12,13]. Several in vitro studies and the hypomineralized dentin phenotype of the *DSPP* null mice confirm the importance of DPP in the dentin matrix [14,15]. Another important function of DPP is its signaling function, which was first demonstrated during embryonic development of the kidneys [16]. We have demonstrated that DPP in the ECM can mediate intracellular signaling events [17,18]. Recently, we have demonstrated that DPP activates Wnt signaling in dental pulp stem cells [19]. Published reports show the importance of Wnt signaling in tooth development using animal models and functional assays [20]. Specifically, our findings indicate that DPP activates Wnt5a expression in dental pulp stem cells. Wnt5a like other Wnt ligands transduces its signal upon binding to various plasma membrane receptors or co-receptor complexes [19]. This results in diverse signaling cascades capable of inducing or repressing β-catenin signaling in vivo and is context dependent. Interestingly, TAZ, a biologically potent transcriptional coactivator with a PDZ-binding motif, serves as a downstream element of the Wnt/β-catenin signaling cascade and is one of the core effectors of the canonical Hippo signaling pathway [21,22]. Interestingly, TAZ expression can be regulated via multiple Hippo-independent mechanisms. In this context, our unexpected observation that stimulation of DPSCs by DPP can upregulate TAZ demonstrates a new signaling function by which DPP promotes the odontogenic differentiation process. A recent review highlights the importance of several signaling pathways on the odontogenic differentiation of DPSCs [23].

In this study, we aim to understand the gene regulatory networks initiated by DPP to promote terminal odontoblast differentiation of DPSCs. Specifically, we report the role of DPP in activating TAZ expression in dental pulp stem cells. We also demonstrate that DPP facilitates the interaction of TAZ with β-catenin to promote gene expression by partnering with transcription factors such as TEA domain (TEAD) family members and TCF/LEF transcription factors during the differentiation of DPSCs into odontoblasts. Thus, DPP, an extracellular matrix protein, plays a pivotal role in activating molecular regulation pathways to promote differentiation of DPSCs into odontoblasts.

## 2. Results

### 2.1. DPP Regulates the Expression of TAZ, a Wnt-Target, in DPSCs

**Figure 1:**
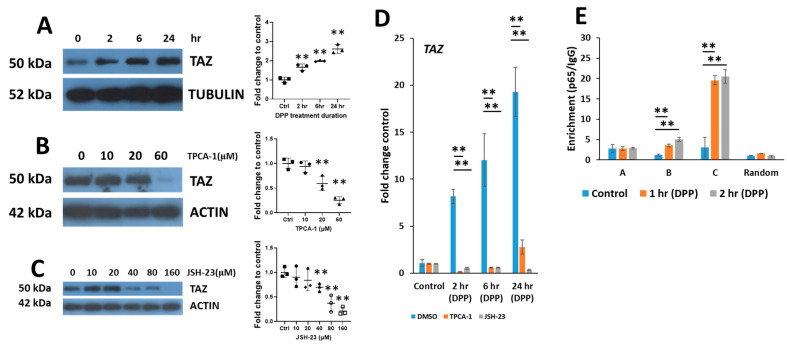
DPP facilitates the activation of TAZ through the NF-kB pathway: (**A**) DPSCs were treated with DPP (500 ng/mL) at various time points (*N* = 3). The expression levels of TAZ were evaluated by Western blotting, with Tubulin as the loading control. (**B**,**C**): DPSCs were pretreated with specific NF-κB signaling inhibitors to assess their effects on TAZ expression in the presence of DPP. (**B**) Cells were pretreated with varying concentrations of TPCA-1 for 1 h before stimulation with DPP and TAZ expression was monitored through Western blotting. (**C**) JSH-23 was administered 1 h prior to DPP treatment, at different concentrations to determine its impact on TAZ expression over a 24 h period. Actin was used as the loading control. (**D**) Quantitative analysis of *TAZ* gene expression at various time points and in the presence of the inhibitors. Fold change in *TAZ* expression is shown relative to 0 h. (**E**) Binding of p65 (RELA), a component of the NF-κB signaling pathway, to specific DNA regions in *TAZ* promoter. Cells were treated with DPP for 1 and 2 h, after which a chromatin immunoprecipitation assay was performed using a specific antibody against p65, alongside a species-matched IgG control. The resulting DNA fragments containing the p65 binding sites were amplified by RT-PCR. All quantitative data are presented as means ± SD of the enrichment ratio (p65/IgG), with an adjacent random DNA region serving as a negative control to validate assay specificity. Statistical significance was indicated, with ** *p* < 0.01.

We have previously shown that DPP activates WNT5A and subsequently stabilizes β-catenin which translocates to the nucleus [19]. To identify the downstream Wnt-targets, activated by DPP in DPSCs, TAZ (also known as WWTR1) was identified as a target of Wnt signaling. To demonstrate induction of TAZ expression, total cellular proteins isolated from DPSCs cultured in growth media with DPP stimulation at various time points were harvested and subjected to Western blotting. The results in Figure 1A showed that TAZ expression steadily increased up to 24 h. However, pretreatment with TPCA-1 and JSH-23, selective inhibitors of NF-κB transcriptional activity, showed a dose-dependent decrease in the levels of TAZ expression (Figure 1B,C). Consistent with the protein data, a significant increase in the expression of *TAZ* gene transcripts were observed from 2–24 h, with a 20-fold increase in *TAZ* expression observed at 24 h. However, pretreatment with NF-κB inhibitors significantly attenuated TAZ expression (Figure 1D).

Bioinformatic analysis of a 2 kb *TAZ* gene promoter revealed the presence of three putative NF-κB DNA binding sites. Analysis of chromatin immunoprecipitation assay using chromatin extracts obtained from confluent DPSCs treated with DPP and performing ChIP-qPCR confirmed p65 interaction at two of these regions. Results in Figure 1E show that the enrichment of DNA binding was observed at binding sites B and C when compared to IgG at both 1 and 2 h of DPP treatment. Interestingly, site C showed up to ~20-fold enrichment. However, enrichment was reduced at site A or by using an adjacent fragment as a control to demonstrate specificity of DNA binding (Figure 1E). The results from the ChIP assay demonstrate that DPP drives TAZ promoter activity by binding to specific NF-κB promoter elements. The assembly of this protein complex is required for *TAZ* gene expression.

### 2.2. DPP-Mediated TAZ Activation Promotes Odontogenic Differentiation

**Figure 2:**
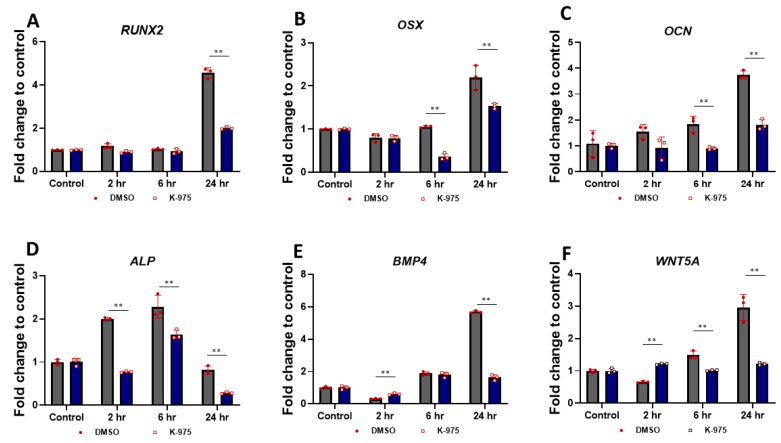
Silencing TAZ expression with K-975 inhibitor attenuates odontogenic gene expression with DPP-stimulation: DPSCs were pretreated with K-975 (50 µM) DMSO (Ctrl) for 1 h, then stimulated with 500 ng/mL of DPP at various time points as indicated. Total RNA was isolated and gene expressions were determined by RT-PCR for *RUNX2* (**A**), *OSX* (**B**), *OCN* (**C**), *ALP* (**D**), *BMP4* (**E**), and *WNT5A* (**F**). Data are presented as average fold induction relative to time 0 ± SD. ** *p* < 0.01.

We next conducted a detailed examination of the role of DPP-mediated TAZ activation during early odontoblast differentiation of DPSCs using quantitative RT-PCR analysis. The results presented in Figure 2A,B,D,E indicate a pronounced increase in the expression levels of early odontoblast differentiation genes, specifically *RUNX2*, *OSX*, and *BMP4*. This increase was observed over a time course from 2 to 24 h, with peak expression levels achieved at 24 h. In addition to these findings, we noted that *ALP* expression reached its highest level at 6 h but subsequently declined at 24 h indicating a temporal regulation of its expression during the odontoblast differentiation process. Alkaline phosphatase is an enzyme that releases inorganic phosphate and is important for the mineralization of the dentin matrix. Furthermore, other odontogenic markers such as *OCN* and *WNT5A* exhibited significantly elevated expression levels at 24 h, as illustrated in Figure 2C,F. In dentin mineralization osteocalcin is important for the alignment of the initial hydroxyapatite crystals. Furthermore, we investigated the specificity of TAZ activity on odontoblast differentiation by pretreating DPSCs with the TAZ inhibitor, K-975. This pretreatment resulted in a notable attenuation of the expression levels of all examined genes (Figure 2A–F), suggesting that TAZ plays a critical role in mediating odontoblast differentiation of DPSCs. The interplay between TAZ and the expression of these key genes highlights the complexity of the regulatory mechanisms during the commitment of DPSCs to the odontoblast lineage.

### 2.3. DPP Facilitates the Interaction of TAZ with Wnt-Signaling Effector β-Catenin in DPSCs

In our previous investigations, we established that DPP plays a crucial role in activating Wnt–β-catenin signaling in DPSCs, leading to β-catenin stabilization. This finding aligns with the existing literature, which identifies Wnt signaling as a significant regulator of TAZ’s nuclear activity. Given this connection, we hypothesized that the activation of TAZ by DPP, along with the stabilization of β-catenin, would promote their translocation into the nucleus to form a protein complex that initiates the transcription of odontoblast specific genes.

**Figure 3:**
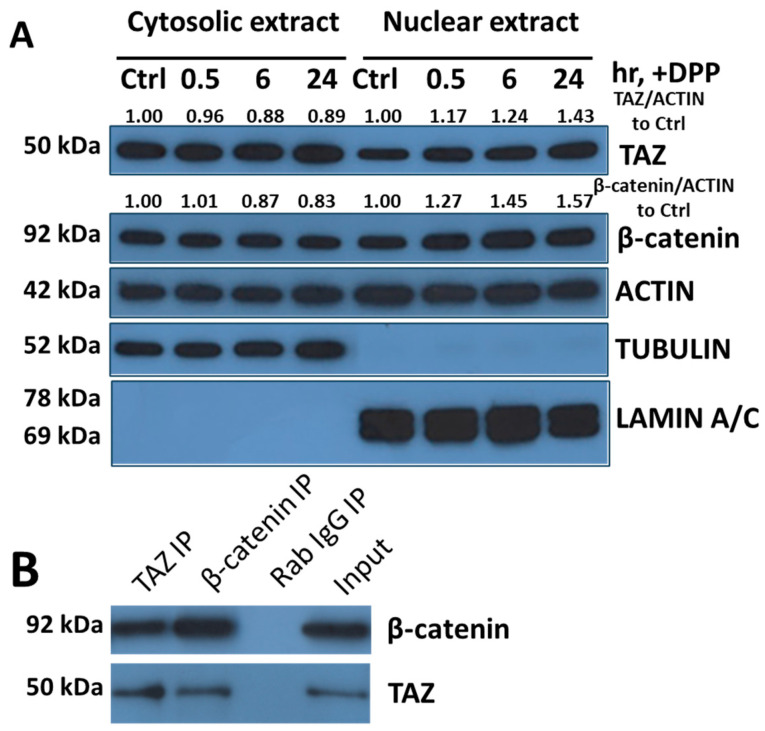
Nuclear translocation of TAZ and β-Catenin and their interaction in DPSCs upon DPP treatment: (**A**) DPSCs were treated with 500 ng/mL of DPP and harvested at 0.5, 6, and 24 h. Cell lysates were prepared and fractioned into cytosolic and nuclear components. The expression levels of TAZ were analyzed using Western blot to assess its subcellular distribution. Actin was used as a loading control for total protein, and the relative band densities of the target protein to 0 h were shown. Additionally, Tubulin and Lamin C served as loading controls and indicators for the cytosolic and nuclear extracts, respectively. (**B**) Immunoprecipitation: DPSCs were treated with DPP for 24 h. Whole cell lysates were extracted and immunoprecipitated using either anti-TAZ antibody, or anti-β-catenin antibody, or an isotype-matched IgG as a negative control. The immunoprecipitants were then analyzed by Western blot using anti-β-catenin and anti-TAZ antibodies to identify their presence in the eluted immunocomplexes.

To test this hypothesis, we conducted a series of experiments in which DPSCs were stimulated with DPP at 0.5, 6, and 24 h. The results, illustrated in Figure 3A, highlight a marked increase in the nuclear translocation of both TAZ and β-catenin in response to DPP stimulation over time. This observation suggests that DPP not only activates the pathways involved but also enhances the localization of these proteins to the nucleus, where they can exert their functions more effectively. To further explore the interaction between TAZ and β-catenin within the nuclear compartment, we performed an immunoprecipitation assay. This assay was designed to elucidate the nature of their interaction and assess whether both proteins could modulate each other’s activity in the nucleus. The findings, presented in Figure 3B, indicate that DPP facilitates the interaction between TAZ and β-catenin, thereby reinforcing the mechanism through which they influence various cellular processes. This crosstalk between TAZ and β-catenin is critical; it underscores their synergistic roles in the development of multiple organ systems, emphasizing the importance of DPP as a mediator of signaling pathways that promote odontoblast differentiation [24].

### 2.4. DPP Stimulation Facilitates Nuclear Colocalization of TAZ and Beta-Catenin

**Figure 4:**
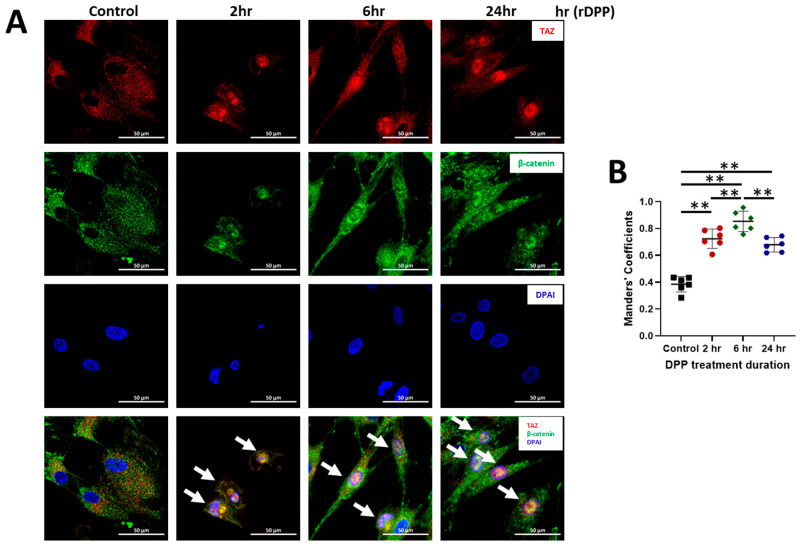
Co-localization of Taz and β-catenin in DPSCs upon DPP treatment: (**A**). DPSCs were seeded onto a cover glass at a density of 1 × 10^4^ cells/cm^2^ and treated with 500 ng/mL of DPP for 2, 6, and 24 h. DPSCs without DPP treatment served as the control. The cells were then fixed using a 10% PBS-buffered formalin for 30 min at 37 °C. Immunofluorescence staining was performed to visualize TAZ (red) and β-catenin (green). Cells were mounted using VECTASHIELD^®^ Antifade Mounting Medium, which also includes DAPI for staining the nuclei (blue). Scale bar = 50 µm. White arrows show cells with strong nuclear TAZ and β-catenin expressions. (**B**) Qualifications of colocalization between TAZ and β-catenin. ** *p* < 0.01.

In order to visualize the movement of TAZ and β-catenin from the cytoplasm into the nucleus with DPP stimulation and their subsequent interaction in the nuclear compartment, we performed imaging by confocal microscopy. The results obtained from confocal microscopy, as illustrated in Figure 4, clearly indicate that prior to DPP stimulation, both TAZ and β-catenin are predominantly localized in the cytosol.

After stimulation, we observed a progressive increase in the nuclear localization of TAZ and β-catenin at the 2, 6, and 24 h time points. This gradual accumulation within the nucleus strongly suggests that DPP plays a critical role in facilitating the translocation of these proteins from the cytoplasm to the nucleus in DPSCs. The nuclear presence of TAZ and β-catenin implies that DPP not only triggers their relocation but also potentially activates transcriptional regulation of gene expression associated with odontoblast differentiation and function

### 2.5. DPP-Mediated Nuclear Translocation of TAZ Activates RUNX2 a Master Transcription Factor of Odontogenesis

A key transcription factor that is activated during the odontogenic differentiation of DPSCs is RUNX2, which plays a critical role in the regulation of genes necessary for tooth/bone development. To investigate the specific function of the transcriptional coactivator TAZ in the odontoblast differentiation process, we generated a stable cell line with a TAZ gene knockout (KO) using the CRISPR-Cas9 gene editing technique. The successful knockout was verified through Sanger sequencing and Western blot analysis, demonstrating the absence of TAZ protein in the edited cell line (Supplementary Figure S1A–D).

**Figure 5:**
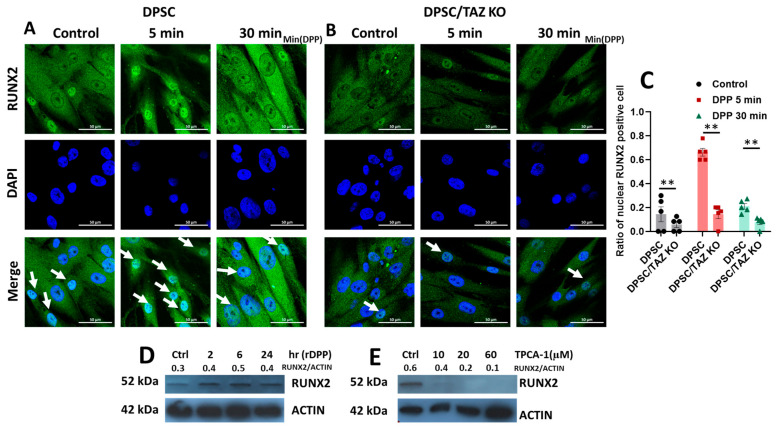
Impaired nuclear translocation of Runx2 in TAZ-KO DPSCs stimulated with DPP: Representative immunofluorescence images were captured at 6 and 12 h after treatment with 500 ng/mL of DPP to evaluate the localization of RUNX2, indicated by green fluorescence. In the DPP-treated DPSCs (**A**) there was a notable increase in the presence of nuclear RUNX2, suggesting that DPP actively promotes the nuclear translocation of RUNX2. (**B**) Conversely, in the TAZ-KO DPSCs, although treated with the same concentration of DPP, this resulted in significant impairment in the nuclear translocation of RUNX2. DAPI staining was used to visualize cell nuclei (blue). Cells without DPP stimulation served as control for both cell types. Scale bar = 50 µm. (**C**) Quantitative comparison of RUNX2 nucleus positive cells upon DPP treatment in DPSC and DPSC/TAZ KO cells. ** *p* < 0.01. (**D**) Western blot analysis using anti-RUNX2 antibody shows that DPP stimulation of DPSCs increased RUNX2 expression. (**E**) Western blot analysis performed on DPSCs treated with NF-κB inhibitor TPCA-1 and probed with anti-RUNX2 antibody shows abrogation of RUNX2 expression.

To assess the implications of TAZ deletion on odontogenic differentiation, we performed immunofluorescence analyses at 6 and 12 h. The results, illustrated in Figure 5A, revealed that upon stimulation with DPP, there was a significant nuclear translocation of the RUNX2 protein that peaked at 6 h post-stimulation in the wild-type DPSCs. However, this nuclear localization of RUNX2 was notably reduced in the TAZ KO cell line in Figure 5B,C, indicating that the presence of TAZ is essential for the full transcriptional activation of RUNX2 during odontoblast differentiation. Further, RUNX2 expression increased with DPP stimulation (Figure 5D). However, TPCA-1, an inhibitor of NF-κB, silenced RUNX2 expression, indicating that DPP-mediated RUNX2 activation is facilitated by NF-κB signaling through TAZ activation in a context-dependent manner (Figure 5D,E). These findings suggest that the transcriptional activity of TAZ is crucial for the differentiation process of DPSCs into odontoblast-like cells.

### 2.6. DPP-Mediated TAZ Activation Regulates Odontogenic Differentiation of DPSCs

**Figure 6:**
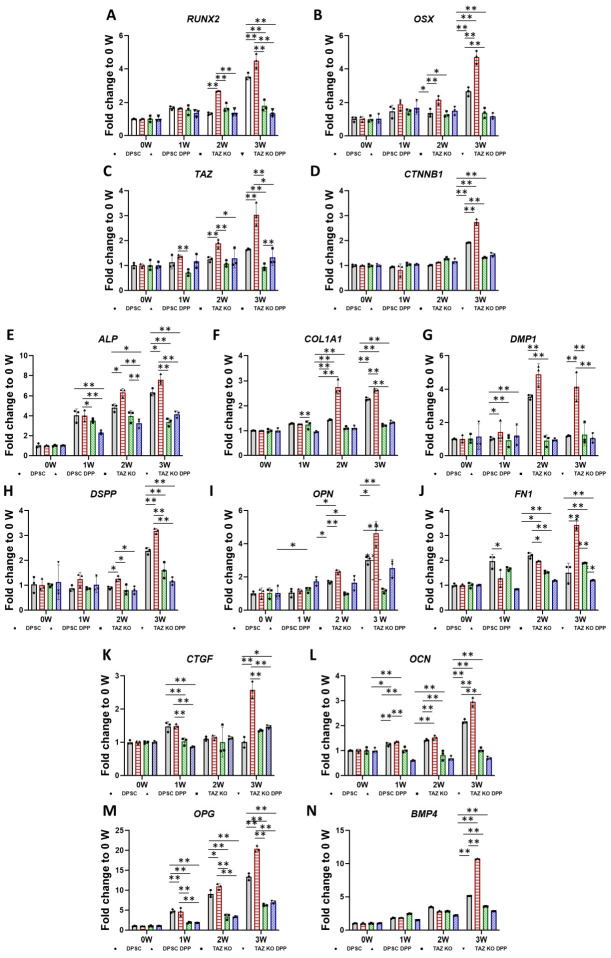
Effect of TAZ signaling on the terminal differentiation of DPSCs into odontoblastic lineage: DPSC and DPSC/TAZ KO cells were cultured under differentiation conditions, with or without DPP, for 1 to 3 weeks. Total RNA was isolated, and quantitative RT-PCR analysis was conducted to assess the expression levels of various odontogenic markers. *RUNX2* (**A**), *OSX* (**B**), *TAZ* (**C**), *CTNNB1* (**D**), *ALP* (**E**), *COL1A1* (**F**), *DMP1* (**G**), *DSPP* (**H**), *OPN* (**I**), *FN1* (**J**), *CTGF* (**K**), *OCN* (**L**), *OPG* (**M**), and *BMP4* (**N**). Fold changes were calculated relative to GAPDH. Note: differentiation markers exhibited lower expression levels in DPSC/TAZ KO cells, even with DPP stimulation, when compared to DPP-treated DPSCs. Significance levels were indicated as ** *p* < 0.01 and * *p* < 0.05. Control without DPP stimulation is indicated as 0 week.

To further assess the odontogenic potential of DPSCs and DPSC/TAZ KO cells, a series of experiments was conducted in which the cells were cultured in differentiation media both with and without 500 ng/mL of DPP over varying time frames of 1, 2, and 3 weeks. After the incubation period, the cells were harvested for analysis. Key odontogenic markers were quantified using real-time PCR, focusing on both “early” and “late” odontogenic differentiation markers. The results, illustrated in Figure 6A–N, indicated a notable upregulation of early odontogenic genes including *RUNX2*, *OSX*, *TAZ*, *CTNNB1*, and *ALP*. Specifically, it was observed that the expression of *BMP4* increased significantly in Figure 6C, showing a more than 10-fold enhancement by the 3-week mark when DPSCs were stimulated with DPP under osteogenic conditions. In contrast, the expression of *BMP4* was markedly reduced in the DPSC/TAZ KO cells, even in the presence of DPP stimulation.

Additionally, the expression levels of various extracellular matrix molecules, such as *COL1A1*, *DMP1*, *OPN*, *FN1*, *OCN*, and *OPG*, were significantly elevated in DPSCs exposed to DPP during the differentiation process, particularly at the 2- and 3-week points, Figure 6B. Moreover, the expression of *CTGF* and dentin *DSPP* showed a substantial increase at the 3-week time point, while lower expression levels were evident in the DPSC/TAZ KO cells. These findings highlight the crucial role of TAZ in the odontogenic differentiation of DPSCs and underscore the impact of DPP stimulation on enhancing key gene expressions associated with dental tissue development.

### 2.7. DPP Stimulation Promotes Interaction of TAZ with TEAD Transcription Factors in DPSCs

TAZ is an essential player in gene regulation, functioning as a coactivator rather than directly binding to DNA. It lacks a traditional DNA binding domain, which enables it to interact with other transcription factors to enhance the expression of downstream target genes. Among these transcription factors, the TEAD family has been identified as the primary interacting partners of TAZ. In our investigation, we sought to determine whether the interaction between TAZ and the TEAD transcription factors is a pivotal factor in facilitating odontogenic differentiation.

**Figure 7:**
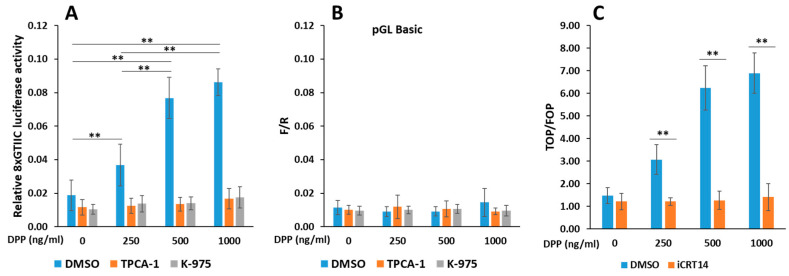
Reporter assays using 8xGTIIC-Lux and TOP-FLASH to demonstrate activation of TAZ and Wnt signaling: DPSCs were seeded at 70% confluence and co-transfected with a TAZ reporter-luciferase vector, 8XGTIIC-Lux, to assess TAZ-dependent transcriptional activity (**A**). Additionally, a pGL basic control reporter vector (**B**), and TOP and FOP reporters (**C**), were combined with a pCMV-Ren for normalization. After 24 h, the cells were treated with DMSO (control), or with inhibitors TPCA1, K-975, or iCRT14 for 2 h. Cells were then stimulated with varying concentrations of DPP (250–1000 ng/mL) and incubated for 48 h. The cells were harvested, lysed, and analyzed using a Dual-Luciferase^®^ Reporter Assay System. The firefly/renilla (F/R) ratios were calculated, and the means and standard deviations were plotted (**A**,**B**, *N* = 9), while means and SDs (*N* = 6) for TOP/FOP ratios were obtained and presented in (**C**). ** *p* < 0.01.

To understand the mechanism by which TAZ functions in terminal differentiation of odontoblasts, we performed a luciferase assay utilizing the 8GTIIC reporter construct. This experiment revealed a significant increase in luciferase activity at DPP concentrations ranging from 250 to 1000 ng/mL compared to the control group. This indicates that higher DPP levels promote greater gene expression potentially via TAZ–TEAD interactions. Interestingly, when we introduced the NF-κB inhibitor TPCA-1 and a specific TAZ inhibitor, K-975, there was a notable reduction in luciferase activity, bringing it back down to baseline levels (as depicted in Figure 7A). This reduction suggests that both TAZ and NF-κB signaling are crucial for the enhanced gene expression observed in the presence of DPP, further underscoring the importance of TAZ’s role in the regulatory mechanisms underlying odontoblast differentiation. For comparative analysis, the control pGL basic vector showed negligible luciferase activity, reinforcing the notion that the observed effects were indeed due to the specific interaction between TAZ and TEAD transcription factors, as illustrated in Figure 7B. Further, a TOPflash/FOPflash luciferase reporter assay was utilized to confirm the activation of the WNT/β-catenin signaling pathway. TOPflash is a luciferase reporter plasmid containing two sets of three copies of the wild-type TCF binding regions. When canonical Wnt signaling is activated, beta-catenin translocates to the nucleus, where it associates with TCF/LEF transcription factors to activate the transcription of Wnt-target genes. As shown in Figure 7C, there is an increase in relative luciferase activity in the presence of DPP; however, this increase is reduced in the presence of the Wnt/β-catenin inhibitor iCRT14. FOPflash serves as a negative control because the TCF binding regions upstream of the luciferase gene are mutated. Consequently, even if beta-catenin translocates to the nucleus, it cannot activate TCF-mediated transcription effectively.

### 2.8. DPP-Mediated TAZ Activation Is Required for the Extracellular Deposition of Mineralized Matrix

**Figure 8:**
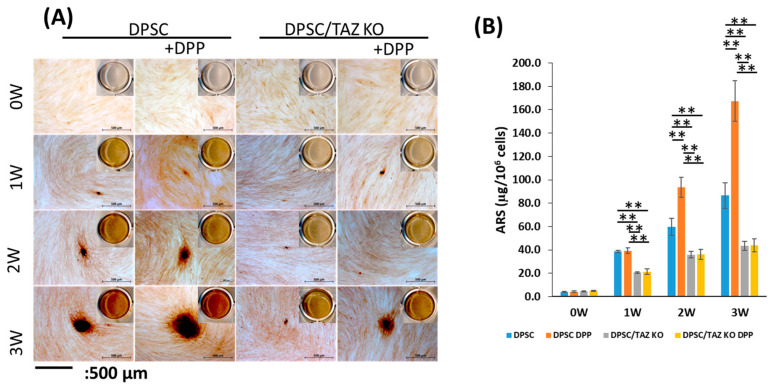
Silencing TAZ in DPSCs impairs their ability to deposit a calcified extracellular matrix. (**A**) DPSCs and DPSC/TAZ KO cells were cultured in 12-well plates at a density of 1.5 × 10^5^ cells per well, using differentiation media with or without DPP for 1 to 3 weeks. Mineralized nodules containing calcium were visualized using Alizarin Red staining. Importantly, DPP-treated DPSCs exhibited robust mineralized nodules; however, no mineralized nodules were noted in the absence of TAZ. A few small mineral nodules were observed with DPP stimulation at the 3-week mark. Insets are scanned images of a well from a 12-well tissue culture plate. Scale bar = 500 µm. (**B**) The quantitative measurement of calcium content in the deposited nodules was conducted by measuring the absorbance of the eluted Alizarin Red stain at 405 nm on a multiplate reader, using a standard Alizarin Red curve. Statistically significant differences are indicated for the 1-, 2-, and 3-week time points ** *p* < 0.01).

To confirm the terminal differentiation potential of DPSCs into functional odontoblasts, we examined whether DPSCs and TAZ KO-DPSCs would deposit a mineralized extracellular matrix under differentiation conditions. This was evaluated by Alizarin Red staining where the dye is incorporated with the calcium containing mineral nodules. The findings, illustrated in Figure 8A, demonstrate a significant difference in mineralization capabilities between the two cell types. Specifically, the DPSCs stimulated with DPP exhibited the formation of large mineralized nodules, indicating robust differentiation and matrix mineralization. In contrast, the TAZ KO-DPSCs, regardless of DPP stimulation, formed smaller nodules, suggesting a compromised ability to mineralize effectively.

Further analysis of the Alizarin Red staining quantification, as shown in Figure 8B, revealed that DPSCs both with and without DPP stimulation accumulated significantly higher levels of calcium deposits by week 3. In stark contrast, the TAZ KO cells demonstrated noticeably smaller deposits with lower calcium content, highlighting a critical functional role for TAZ in the functional differentiation of DPSCs into odontoblasts. These results underscore that TAZ is a major player for lineage specific differentiation of DPSCs into functional odontoblasts.

### 2.9. DPP Is Required for TAZ- and β-Catenin-Mediated Odontoblast Differentiation In Vivo

**Figure 9:**
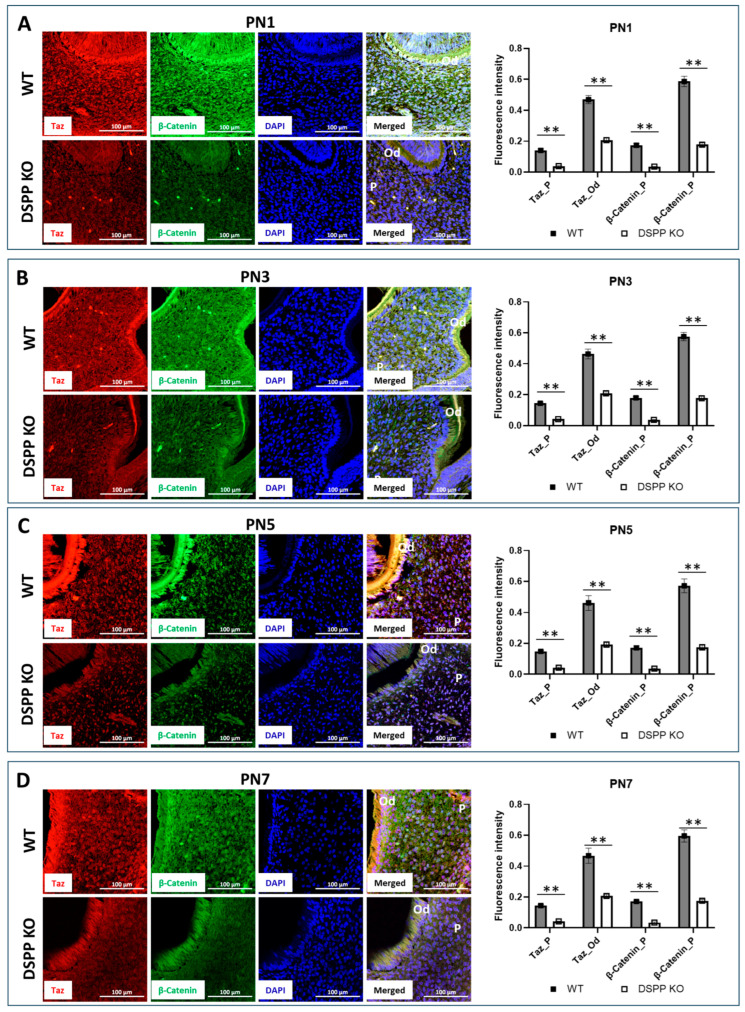
Localization of Taz and β-catenin in mouse molar: Representative immunostainings and quantification of fluorescence intensity for TAZ (red) and β-catenin (green) performed on day 1 (**A**), 3 (**B**), 5 (**C**), and 7 (**D**) mouse head sections from both wild-type and DSPP knockout mice. Nuclei were detected with DAPI. The anatomical regions of interest are labeled, as “P” denoting the pulp and “Od” representing the odontoblasts. PN: postnatal day. Scale bar = 100 µm. ** *p* < 0.01.

To investigate the role of DPP in mediating TAZ-dependent odontoblast differentiation in vivo, we utilized the well-characterized Dentin Sialophosphoprotein Knockout (DSPP-KO) mouse model. DSPP is a crucial matrix protein involved in matrix mineralization and the dentin phenotype in DSPP null mice shows impaired dentin mineralization. Immunofluorescence analysis performed on postnatal day 1 (Figure 9A), 3 (Figure 9B), 5 (Figure 9C) and 7 (Figure 9D) mice show that the expression levels of TAZ and β-catenin are highly reduced in DSPP-KO mice compared to wild-type controls at the same age. This notable decrease aligns with previously published studies highlighting the essential role of DPP in regulating mineralization processes within dental tissues. These results suggest the expression of TAZ and β-catenin and their interaction in the presence of DPP confirms the critical role of DPP in promoting odontoblast differentiation of dental pulp cells. This interaction is essential for proper dental development, as it influences the cellular mechanisms that lead to the formation of functional odontoblasts, which are responsible for dentin production.

## 3. Discussion

Odontogenesis is a multistep process initiated by the ectoderm and the cranial neural-crest derived ectomesenchyme [1]. The neural-crest mesenchyme migrates from the crest of neural ectoderm and interacts with the signals from the oral ectoderm. This process activates a signaling cascade leading to the formation of the dentin–pulp complex. The cellular processes involve cell proliferation, differentiation, and migration. Several signaling pathways are activated during this process. In vivo studies using animal models have shown that YAP (Yes-associated protein) and TAZ (transcriptional coactivator with PDZ-binding motif),effectors of the hippo pathway have a critical function in tooth morphogenesis [25]. Both YAP and TAZ are expressed during tooth development; however, YAP expression is restricted to the odontogenic epithelium from the bud stage to the bell stage. TAZ expression was mainly observed in odontoblasts involved in dentin formation and in pre-ameloblasts opposing the odontoblasts [26]. This expression profile restricted to the developing mesenchymal cells strongly suggests that TAZ signaling is necessary for odontoblast differentiation, dentin matrix formation, and mineralization.

The extracellular dentin matrix contains several noncollagenous proteins [27]. DPP is nature’s most acidic protein synthesized by the odontoblasts, therefore linked to matrix mineralization [12]. We and others have shown that besides matrix mineralization, DPP functions as a signaling molecule as DPP stimulation causes release of intracellular Ca^2+^ in mesenchymal precursor cells [28,29]. This intracellular calcium flux triggers several cellular responses. Previous studies from our lab have implicated that DPP in the matrix can activate Wnt5a-mediated signaling events to promote the differentiation of DPSCs into functional odontoblasts. Interestingly, this differentiation process was facilitated by the expression of Wnt-target genes in a β-catenin-dependent manner [19]. Recent studies show that TAZ activation is a downstream effector of Wnt signaling [24,30]. This signal stabilizes TAZ in the cytoplasm and promotes its nuclear translocation. TAZ functions as an important transcriptional coactivator as it lacks DNA-binding domains. They partner with transcription factors to modulate the expression of their target genes. Thus, we propose a new signaling function activated by DPP that regulates TAZ/Wnt-related signaling pathways to promote differentiation of dental pulp stem cells into functional odontoblasts.

Earlier we demonstrated that DPP facilitated the interaction of Wnt5a with receptor Frizzled 5 and LRP6 to induce nuclear translocation of β-catenin [19]. When Wnt signaling is activated, TAZ is released from the complex and translocates to the nucleus, where it can partner with β-catenin to activate target genes. In this study, we show specifically that DPP facilitates the interaction between TAZ and β-catenin in the nucleus of DPSCs. Both TAZ and β-catenin are two key proteins with overlapping and distinct functions as β-catenin is a transcription factor while TAZ is a transcriptional coactivator. These proteins are expressed in a specific temporal and spatial manner during tooth development [26,31,32,33,34]. TAZ primarily interacting with the Hippo signaling pathway and β-catenin with the Wnt signaling pathway. Interaction between TAZ and β-catenin influences each other’s activity and stability. This interplay between TAZ and β-catenin can influence various cellular processes during tooth development such as tooth germ formation, differentiation of epithelial and mesenchymal tissues, and overall tooth morphogenesis.

We have previously reported that expression of differentiation and matrix mineralization proteins such as RUNX2, OSX, ALP, and OCN, were activated by DPP stimulation and were inhibited by iCRT14, a specific β-catenin transcription inhibitor [35]. In this study, the same genes were also inhibited by K795, a TAZ/TEAD inhibitor [36]. Using TAZ KO-DPSCs we demonstrated that DPP-mediated TAZ activation was responsible for the regulation of several odontoblast differentiation transcripts such as DMP1, FN (fibronectin), DSP, OPN, CTGF, OPG and early transcripts such as Runx2 and BMP4. The expression of ALP, a phosphatase involved in phosphate homeostasis, decreased in TAZ-KO cells. In the presence of DPP, an increase in the expression of the differentiation markers was observed from 1–3 weeks in differentiation media. Terminal differentiation of odontoblasts was confirmed by the functional Alizarin Red assay. Corresponding to the gene expression pattern, matrix mineralization increased from 1 to 3 weeks as shown by the increased calcium deposition.

TAZ is a transcriptional coactivator, and has no DNA binding domain; therefore, it binds to other transcription factors to stimulate downstream target gene expression. TEAD family transcription factors are the major TAZ interacting transcription factors in DPSCs and we demonstrate that TAZ indeed binds to the transcription factor TEAD when stimulated with DPP. In addition, Wnt specific transcriptional activity was also activated by DPP as demonstrated by promoter luciferase assay. Indeed, many TAZ-dependent odontogenic differentiation targets are also activated by the Wnt/β-catenin signaling pathway. Cross-talk between β-catenin and TAZ signaling pathways as seen in this study can activate the differentiation of DPSCs into odontoblasts.

Transcription factor Runx2 plays a key role in odontoblast differentiation, cartilage hypertrophy, and vascular invasion of bone [37,38,39,40]. Immunofluorescence studies demonstrate activation of RUNX2 at 6 h as demonstrated by its nuclear translocation with DPP. In TAZ-silenced DPSCs, nuclear translocation was abrogated. Further, activation of RUNX2 by DPP was inhibited by K-975 a strong and selective TEAD inhibitor which inhibits TAZ–TEAD protein–protein interactions, indicating that RUNX2 is a downstream gene target of TAZ. Additionally, RUNX2 promoter contains β-catenin/TCF binding sites and can also be activated by Wnt/ β-catenin signaling [41].

Using human intrahepatic cholangiocarcinoma cell lines, RNA-seq analysis demonstrated that YAP/TAZ regulated a set of genes significantly overlapping with those controlled by β-catenin, e. g., 1003 genes overlapping between a total 2519 YAP/TAZ regulated genes and 3338 β-catenin regulated genes [42]. We had previously shown that DPP stimulation activated NF-kB target genes as well as WNT5A/β-catenin pathway odontogenic/osteogenic target genes [43]. In this study we confirmed that DPP activates both these signaling pathways using TOP/FOP and luciferase assays. Thus, it is possible that both TAZ and β-catenin synergistically regulate odontogenic differentiation of DPSCs by activating Runx2.

During odontogenesis, when TAZ is overexpressed, it activates the TGF-beta signaling pathway and suppresses the proliferation of dental pulp stem cells [25]. Tian et al. [44] showed that miR-584 directly binds to TAZ mRNA 3′UTR to suppress its translation, thereby inhibiting the proliferation of DPSCs. Decrease in TAZ protein levels was detected in the aging human dental pulp tissue with the upregulation of miR-584.

Homozygous TAZ null mice show elevated blood urea nitrogen, partial postnatal lethality due to an unknown reason, premature death, glomerulocystic kidney disease, and minor skeletal defects [45]. On the other hand, osteoblast specific conditional Taz knockout mice (Taz^Oc-KO/Oc-KO^) had low bone formation rate with significant bone mass through reduction of TAZ-mediated osteoblast differentiation with an increase in osteoclast activity [46]. Interestingly, DSPP (precursor protein of DPP) null mice developed teeth with a widened pre-dentin zone and defective dentin mineralization, a phenotype similar to human dentinogenesis imperfecta Type III [14]. In this study using DSPP-KO mice we show by temporal and spatial analysis that the absence of TAZ and β-catenin in the odontoblasts and dental pulp cells resulted in impaired dentin mineralization when compared with the wild type mice. Additional published studies revealed mineralization defects in dentin, alveolar and calvaria bones, and suture formation during development [47,48]. These data clearly suggest that TAZ activation mediated by DPP can affect both osteoblast and odontoblast differentiation and matrix mineralization.

We had shown earlier that DPP activates NF-κB, resulting in the transcriptional regulation of early markers that promote odontogenic differentiation of DPSCs. Interestingly, *TAZ* proximal promoter contains three putative NF-κB binding sites, of which two show enhanced binding with DPP stimulation. Similarly, NF-kB binds to WNT5A promotor region and activates its expression and facilitates β-catenin-mediated odontoblast differentiation. In this study, we demonstrate that DPP stabilizes both β-catenin and TAZ in the cytoplasm and facilitates their nuclear translocation. In the absence of a stimulus, TAZ protein can be phosphorylated by LATS on the serine residues in the HXRXXS motif and can lead to enhancement of 14-3-3 binding, resulting in cytoplasmic retention [49].

Although we demonstrate that both WNT5A and TAZ expression were upregulated by DPP through NF-κB signaling pathway during odontoblast differentiation, there are other factors that might contribute to fine-tune the expression of these proteins. Of these non-coding RNAs, specifically miRNAs can regulate translation by targeting 3′UTR of TAZ or WNT5A mRNA. However, miRNA-dependent regulation of DPP is largely an understudied area.

## 4. Materials and Methods

### 4.1. Cell Culture

Dental pulp stem cells (DPSCs), a kind gift from Dr. Songtao Shi at the University of Pennsylvania [8,9], were cultured in growth media αMEM (Life Technologies Corporation, Carlsbad, CA, USA) with 15% FBS and 100 units/mL of penicillin, 100 μg/mL of streptomycin, and 0.25 μg/mL of Amphotericin B (Life Technologies Corporation) at 37 °C, 5% CO_2_ in a humidified incubator. At 80% confluency, the cells were trypsinized and split at 1:3 ratio (one passage). For all experiments, cells from passages 4 to 10 were used.

### 4.2. CRISPR-Cas9-Based Knockout of TAZ in DPSCs

lentiCRISPR vector (LV01/TAZ) containing predesigned CRISPR gRNAs targeting *TAZ* was purchased from Sigma-Aldrich (HSPD0000076623, Sigma-Aldrich, Inc., St. Louis, MI, USA). Knockout of *TAZ* in DPSCs was performed as recommended by the manufacturer and described previously [19]. Briefly, the LV01/TAZ, together with the psPAX2 (Addgene, Watertown, MA, USA), pMD2.G (Addgene), and pHPV17 plasmids were transfected in 293FT cells (Life Technologies Corporation) using Lipofectamine 2000 (Thermo Fisher Scientific Inc., Waltham, MA, USA). After 48 h, virus-containing supernatants were collected and viral particles were obtained by centrifugation at 1000× *g* for 10 min, followed by 75,000× *g* for 4 h at 4 °C. The viral particles were then used to infect DPSCs in the presence of 10 µg/mL polybrene in DMEM basal media (Life Technologies Corporation). After 72 h, 1 µg/mL puromycin was added to the DPSC culture for 5 days. The puromycin resistant cells were pooled, and chromosomal DNA was extracted from the cells with Direct PCR Lysis Reagent (Viagen Biotech, Inc., Los Angeles, CA, USA). The DNA region flanking targeted gDNA was PCR-amplified using Platinum™ Taq DNA Polymerase (Life Technologies Corporation) with primer pair (WWTR1_22&3_F_814:-GCA GAA GAT GAA TCC GGC CT-;WWTR1_22&3_R_1270:-TGC CTT CTT CGG CTC CAG GCT GAC TTA C-). PCR products were cleaned with QIAquick Gel Extraction Kit (QIAGEN, Germantown, MD, USA) and analyzed by Sanger sequencing at Research Resources Center/University of Illinois Chicago to confirm the targeted mutation. Subsequently, knockdown of cellular TAZ protein was demonstrated by Western blot. These cells were thus designated as DPSC/TAZ KO and used in subsequent experiments.

### 4.3. Immunocytochemistry

DPSCs (50,000) were seeded on a cover glass (D = 12 mm, Thermo Fisher Scientific Inc.) and placed in a 24-well plate. After cell attachment, fresh growth media with 5% FBS were added and incubated for 12 h. DPP at 500 ng/mL (based on previous studies [28,43]) was then added. After 0, 2, 6, and 24 h of treatment, the cells were washed with PBS and then fixed with 10% neutral formalin for 1 h at room temperature (RT). The cells were then further washed 3 times with PBS, treated with 0.5% BSA, 0.3% Glycine in PBS at RT for 1 h, and permeabilized with 0.25% Triton X-100 in PBS for 30 min at RT. After washing with PBS, the cells were blocked with 10% BSA in PBS, followed by incubation with an anti-TAZ antibody (4883S, Cell Signaling Technology Inc., Danvers, MA, USA) and an anti β-catenin antibody (05-665, Sigma-Aldrich) in PBS with 1% BSA at 4 °C overnight. The cells were then washed with PBS, incubated with Alexa Fluor secondary antibody (Thermo Fisher Scientific Inc.), and mounted and imaged with a light microscope (Zeiss Axio Observer D1, Carl Zeiss Microscopy LLC, White Plains, NY, USA) as published [19]. The images were analyzed with ImageJ (Ver 1.48) to obtain colocalization coefficients.

Similarly, DPSCs and DPSC/TAZ KO cells were stimulated with DPP at 500 ng/mL for the indicated time duration and processed with an anti-RUNX2 antibody (Abcam Inc., Waltham, MA, USA.) followed by Alexa Fluor secondary antibody.

### 4.4. Subcellular Fractionation and Western Blotting

To obtain cytosolic and nuclear fractions, DPSCs were cultured in growth media for 24 h, then changed to fresh growth media with 5% FBS and incubated for 12 h. The cells were then stimulated with 500 ng/mL of DPP and harvested at 0, 0.5, 6 and 24 h. They were washed with cold PBS and lysed in ice cold lysis buffer and scraped with a cell scraper. Cell fractionation was conducted with NE-PER Nuclear and Cytoplasmic Extraction Kit (Thermo Fisher Scientific Inc.) following the manufacturers protocol with the addition of phosphatase and proteinase inhibitor cocktail (MilliporeSigma, Burlington, MA, USA). The concentration of the proteins was determined by Bio-Rad Protein Assay Dye Reagent Concentrate (Bio-Rad Laboratories Inc., Des Plaines, IL, USA) with BSA as a standard.

For Western blot analysis, 10 µg protein samples were separated on a 10% SDS-PAGE and transferred to a PVDF membrane. The membrane was washed with PBS, blocked with 5% milk in PBS for 1 h RT. Next, the membrane was incubated with anti-TAZ (4883, Cell Signaling Technology Inc.) and anti-β-catenin antibody (PLA0230, MilliporeSigma) in 1% milk overnight at 4 °C. After washing, the membrane was then incubated with an HRP-conjugated secondary antibody (Cell Signaling Technology Inc.) for 2 h at RT followed by washing with PBS. Protein bands were visualized using Thermo Scientific™ Pierce™ ECL Western Blotting Substrate (32106 Thermo Fisher Scientific Inc.) and imaged. The membranes were then stripped with Restore PLUS Western Blot Stripping Buffer (Thermo Fisher Scientific Inc.) and probed with actin, while tubulin (T5168, MilliporeSigma) and lamin A/C (4777, Cell Signaling Technology) were used as protein loading controls for cytoplasmic and nuclear fractions, respectively. The developed films were scanned, and images were analyzed with ImageJ (Ver 1.48) to obtain the densities of the target bands.

### 4.5. Coimmunoprecipitation and Western Blotting

DPSCs were seeded, and after cell attachment, fresh growth media containing 5% FBS were added and incubated for 12 h. Cells were then treated with DPP (500 ng/mL) for 6 h. The cells were washed with cold PBS and then lysed using Cell Lysis Buffer (Cell Signaling Technology Inc.) containing proteinase and phosphatase inhibitors and centrifuged at 14,000 rpm for 20 min. Then 1 mg of whole cell lysates were incubated with 5 µg of anti-CTNNB1 antibody (C2206, Sigma-Aldrich), anti-TAZ antibody (4883S, Cell Signaling Technology Inc.), and anti-Rab IgG (ab27478, Abcam Inc.) at 4 °C overnight. Then 20 µL of Protein A/G Magnetic Agarose Beads (Thermo Fisher Scientific Inc.) were added and incubated for 2 h at RT. The magnetic beads with the immune complex were collected by using a PureProteome Magnetic Stand (Sigma-Aldrich), followed by PBS wash and eluted with 0.3 M Glycine buffer (pH 2.2), and the eluates were neutralized with 2 M Tris-pH 8.8. The detection of β-catenin and TAZ in the immune complexes was conducted as described above. To distinguish detection between TAZ and IgG heavy chain with similar mobility on SDS-PAGE gel, a specific HRP conjugated anti-TAZ antibody (sc-518026 HRP, Santa Cruz Biotechnology Inc., Dallas, TX, USA) was used to avoid IgG detection.

### 4.6. Quantitative Real-Time PCR (qRT-PCR)

DPSCs were treated with 500 ng/mL of DPP and harvested at the indicated time points as described above. The total RNA was obtained from cells using TRIzol Reagent (Life Technologies Corporation) following the manufacturer’s instructions. cDNAs were synthesized using 2 µg of total RNA and Maxima First Strand cDNA Synthesis Kit with dsDNase (Thermo Fisher Scientific Inc.) in a 20 µL reaction. Quantitative real-time PCR was conducted using FastStart™ Universal SYBR^®^ Green Master (Roche Diagnostics, Indianapolis, IN, USA) with the STEP one plus instrument (Thermo Fisher Scientific Inc). The gene expression levels were estimated by the 2^−ΔΔCT^ method with GAPDH gene expression level as an internal control. Primers were synthesized by IDT (Integrated DNA Technologies, Inc. Coralville, IA, USA). The primer sequences are listed in Table 1.

### 4.7. Transient Transfection and Promoter Analysis by Luciferase Assay

Luciferase reporter assays were performed using constructs containing genomic regions with putative TEAD binding sites in DPSCs. One day prior to transfection, 1.5 × 10^5^ DPSCs were seeded in one well of a 12-well tissue-culture plate (BD Biosciences, San Jose, CA, USA). After 12 h, fresh OPTI-MEM media were added and co-transfections were conducted using the transfection vector mixture (total 275 ng plasmid DNA in 25 μL OPTI-MEM media) containing 250 ng of each TAZ/TEAD reporter luciferase construct (8xGTIIC-luciferase, # 34615, Addgene), 250 ng of empty pGL Basic reporter construct (Promega, Madison, WI, USA), or 250 ng of TOPFLASH and FOPFLASH reporter constructs (kind gifts from Dr. Lyndon F. Cooper) along with 25 ng of CMV/Renilla luciferase vector (Promega) serving as the transfection efficiency controls. Then 1.5 μL of Lipofectamine 2000 (Life Technologies Corporation) was added to the mixture, and co-transfections were conducted according to the manufacturer’s instructions. After 16 h, the cells were cultured with fresh a-MEM containing 5% FBS for 12 h, then TPCA-1 (10 µM, Sigma-Aldrich), K-975 (50 µM, Sigma-Aldrich) or iCRT14 (25 µM, Sigma-Aldrich) for 1 h were added for an additional 1 h (an equal amount of DMSO solvent was used in the control group), followed by the addition of DPP. After incubation for 48 h, cell lysates were prepared using passive lysis buffer (Promega). Luciferase activities in the lysates were measured using a dual luciferase assay system (Promega) with a plate reader (Synergy 2, BIOTEK, Winooski, VT, USA).

### 4.8. Chromatin Immunoprecipitation (ChIP)

Bioinformatics analysis (https://jaspar.elixir.no/, accessed on 12,October 2020) was used to identify the NF-ĸB p65 binding consensus sequence GGGNNNNNCC in the 2 kb promoter regions in the 5′-UTR region (>5′ Flanking sequence chromosome: GRCh38:3: 149,658,026–149,660,025: −1) of the *TAZ* gene (ENST00000360632.8 WWTR1-201, GRCh38:3: 149,517,235–149,658,025: reverse strand). Relative to GRCh38: 149,660,025 at −1, the positions of putative NF-ĸB p65 binding were site C: −1797 to −1785; B: −858 to −846; C: −527 to −515, while region −183 to −50 (random control) contained no such site. To test the possibility that DPP activates *TAZ* gene expression by binding of NF-κB p65 subunit to the putative promoter elements of genes involved in odontogenic differentiation, specific primer pairs flanking each of three putative binding elements were designed and used to detect the DNA fragments in the eluted immune complex following CHIP assay by RT-PCR. A primer pair amplifying a random region adjacent to the NF-κB promoter binding element was synthesized and designated as a negative control. Then 2 × 10^6^ DPSCs were treated with DPP (500 ng/mL) for 1 or 2 h and washed with cold PBS once and fixed with PBS neutralized 10% Formalin for 15 min at RT with gentle shaking. Chromatin immunoprecipitation (ChIP) assays were performed with a commercially available ChIP-IT high sensitivity kit following the manufacturer’s protocol (Active Motif North America, Carlsbad, CA, USA). A total of 5 µg of an anti-p65 antibody (ab16502, Abcam Inc.) was used in immunoprecipitation, while 5 µg of Rab IgG (ab27478, Abcam Inc.) served as a negative control. Target DNA fragments were amplified by real time-PCR using specific primers. Relative amounts of the DNA fragments were calculated as percentages of the input DNA that was used in the ChIP assay. The primer sequences are listed in Table 2.

### 4.9. Differentiation Assay Using Alizarin Red Staining

DPSCs and DPSC/TAZ KO cells were cultured in growth media until 80% confluent. Then they were cultured in osteogenic differentiation media (growth medium containing 10 mM β-glycerophosphate (Thermo Fisher Scientific Inc.), 0.50 mM ascorbic acid (Sigma-Aldrich), and 10 nM dexamethasone (Sigma-Aldrich) with or without DPP (500 ng/mL) for 7, 14, and 21 days. At each time point, the cells were washed with PBS and fixed in 10% neutral formalin at 4 °C for 4 h. Cells were then stained with 2% Alizarin Red S (Sigma-Aldrich) for 30 min, then rinsed with water. The plates were scanned to visualize the overall staining pattern and high magnification images were obtained with a light microscope (Zeiss Observer D2, Carl Zeiss Microscopy LLC,.). The bound Alizarin Red S was extracted by 10% acetic acid at 85 °C for 10 min. The extracts were neutralized by adding 10% ammonium hydroxide at 1/3 volume of the initial 10% acetic acid. The Alizarin Red S concentrations were measured by ABS_405nm_ with Alizarin Red S as a standard using a plate reader (Synergy 2, BIOTEK).

### 4.10. Immunohistochemical Analysis of TAZ and β-Catenin in WT and DSPP-Null Mice

Mouse breeding colony management and experimental procedures were approved by the Institutional Animal Care and Use Committee (IACUC) at the University of Illinois Chicago and in compliance with the ARRIVE (Animal Research: Reporting of In vivo Experiments) guidelines. Postnatal day (PN) 1, 3, 5 and 7 mouse heads from WT and DSPP-null mice were obtained and fixed in PBS buffer neutralized with 10% formalin for 12 h at RT, then washed 3 times with water, followed by dehydration using gradient ethanol exchange, transferred to Xylene, and then embedded in paraffin. Then 5 µm sections were obtained with a microtome (Leica RM2135, Leica Biosystems, Deer Park, IL, USA). The sections were de-paraffinized and rehydrated with gradient ethanol exchange, followed by PBS washing 3 times. Antigen retrieval was conducted in citric acid buffer (10 mM Citric Acid, 0.05% Tween 20, pH 6.0) at 95 °C for 30 min. After washing with PBS 5 times, the sections were treated with 0.25% TritonX-100 in PBS for 30 min, followed by a PBS rinse. Then the sections were blocked with 10% BSA in PBS at RT for 1 h, then incubated with TAZ (4883S Cell Signaling Technology Inc.), β-catenin (05-665, MilliporeSigma) antibodies in 1% BSA-PBS at 4 °C overnight. After PBS washing, the sections were incubated with goat anti-rabbit secondary antibody Alexa Fluor 594 (A11012, Life Technologies Corporation) and goat anti-mouse secondary antibody Alexa Fluor 488 (A11029, Life Technologies Corporation) at RT for 1 h. Then, the PBS-washed sections were mounted with Antifade Mounting Medium with DAPI (H-1200-10, Vector Laboratories, Inc., Newark, CA, USA), and images were taken with a Zeiss confocal microscope at the RRC of UIC. The images were analyzed with ImageJ (Ver 1.48) to obtain fluorescence intensity.

## 5. Conclusions

**Figure 10:**
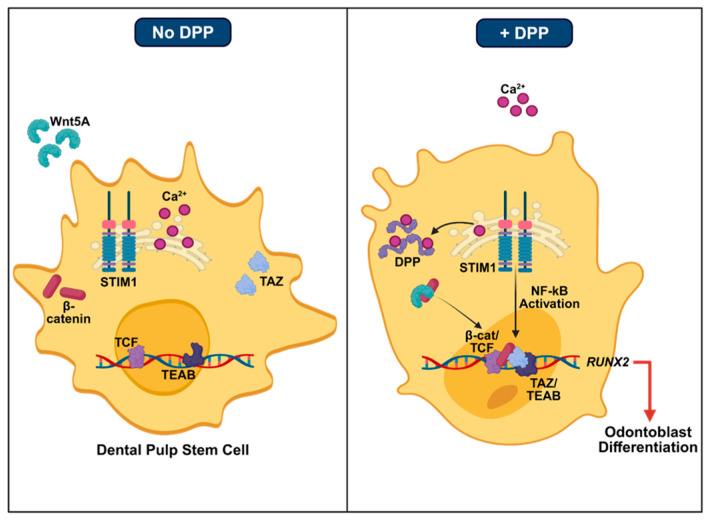
Proposed model. A model depicting the proposed mechanism by which DPP in the extracellular matrix can promote differentiation of dental pulp stem cells into odontoblasts by activating TAZ which translocates to the nucleus. DPP also activates Wnt signaling to induce nuclear translocation of β-catenin. In the nucleus, interaction of TAZ and β-catenin along with other transcription factors form complexes that modulate transcription of target genes resulting in the odontoblast-specific lineage differentiation of dental pulp stem cells.

In this study, we provide compelling evidence that DPSCs when stimulated with DPP lead to the activation of TAZ, a crucial transcriptional effector that functions downstream of the Wnt signaling pathway. This activation is essential for promoting lineage specific differentiation of DPSCs into functional odontoblasts. The role of DPP in activating TAZ signaling was confirmed in the TAZ KO DPSCs. Silencing TAZ led to a significant decrease in the expression of genes associated with matrix mineralization. Further, our findings indicate that the TAZ-mediated activated odontogenic genes possess conserved TEAD binding domains and the TAZ–TEAD protein–protein interactions facilitated odontoblast differentiation, suggesting a robust regulatory mechanism. Interestingly, DSPP KO mice exhibited notable downregulation of TAZ expression in the odontoblasts and dental pulp, resulting in impaired dentin formation. Additionally, our research demonstrates that DPP also activates the Wnt signaling pathway, which is critical for the odontogenic differentiation of DPSCs. Within this pathway, β-catenin emerges as a pivotal factor that influences the differentiation process. Notably, we observed that TAZ interacts with β-catenin, and together they synergistically cooperate to enhance odontogenesis and matrix mineralization. Overall, an intricate crosstalk mediated by DPP between TAZ and Wnt–β-catenin signaling pathways facilitates the functional odontogenic differentiation of DPSCs (Figure 10). Understanding this molecular mechanism would be beneficial in regenerative dentistry.

## Figures and Tables

**Table 1:** DNA oligoes for quantitative PCR.

Gene Name	Accession	Sequence
ALP	NM_001127501	AAC ATC AGG GAC ATT GAC GTG
		GTA TCT CGG TTT GAA GCT CTT CC
OSX	AF477981	GCC AGA AGC TGT GAA ACC TC
		GCT GCA AGC TCT CCA TAA CC
RUNX2	NM_001015051	TGG TTA CTG TCA TGG CGG GTA
		TCT CAG ATC GTT GAA CCT TGC TA
FN1	NM_212482.2	CAG TGG GAG ACC TCG AGA AG
		GTC CCT CGG AAC ATC AGA AA
COL1A1	NM_000088	GAG GGC CAA GAC GAA GAC ATC
		CAG ATC ACG TCA TCG CAC AAC
OPN	NM_000582	AGG AGG AGG CAG AGC ACA G
		GAG ATG GGT CAG GGT TTA GC
OPG	NM_002546	CAA AGT AAA CGC AGA GAG TGT AGA
		GAAGGTGAGGTTAGCATGTCC
OCN	NM_199173	CAC TCC TCG CCC TAT TGG C
		CCC TCC TGA TTG GAC ACA AAG
DMP1	NM_004407.3	AAT TCT TTG TGA ACT ACG GAG GG
		CAC TGC TCT CCA AGG GTG G
VEGFA	NM_001171627	AGG GCA GAA TCA TCA CGA AGT
		AGG GTC TCG ATT GGA TGG CA
WNT5A	XM_011534088	GCC AGT ATC AAT TCC GAC ATC G
		TCA CCG CGT ATG TGA AGG C
GAPDH	NM_001357943	GGA GCG AGA TCC CTC CAA AAT
		GGC TGT TGT CAT ACT TCT CAT GG
TAZ	NM_001168278	GAT CCT GCC GGA GTC TTT CTT
		CAC GTC GTA GGA CTG CTG G
BMP4	NM_001202	TAG CAA GAG TGC CGT CAT TCC
		GCG CTC AGG ATA CTC AAG ACC
CTNNB1	NM_001098209	CAT CTA CAC AGT TTG ATG CTG CT
		GCA GTT TTG TCA GTT CAG GGA
DSPP	NM_014208	GTT GGA CAC AGC AAT ACA GGT
		TCC TTT TGA GTC ACT GCC AT
CTGF	NM_001901	CAG CAT GGA CGT TCG TCT G
		AAC CAC GGT TTG GTC CTT GG

**Table 2:** DNA oligoes for CHIP quantitative PCR.

WWTR1-p65-A-F	AAGCAGCCTCTCGTGGAAAA
WWTR1-p65-A-R1	GCATCTGCATTCCTTTGAGTTT
WWTR1-p65-B-F	CTACTTCCAGCCACCTGCTC
WWTR1-p65-B-R2	CAGGCCACTAGAGATTGAGAAG
WWTR1-p65-C-F	ACTCCATCCTGGGCAATAGA
WWTR1-p65-C-R	ACTCCCTGAATGGCTGCATC
WWTR1-NON-F1	AAAGTACCCATCACGCCCAG
WWTR1-NON-R1	GCCCGAAAGTTGAGCTGTTG

## Data Availability

The sequence data that support the findings of this study have been deposited in the GenBank repository with the accession number PV933766. The additional data that support the findings of this study are available from the corresponding author upon reasonable request.

## Supplementary Materials

The following supporting information can be downloaded at: https://www.mdpi.com/article/10.3390/ijms27104599/s1.
