# Supplementary material for: DPP-Mediated Interaction of TAZ/β-Catenin Promotes the Differentiation of DPSCs into Odontoblasts
Source: Int J Mol Sci. 2026 May 20;27(10):4599. doi: 10.3390/ijms27104599 (PMC13207296; doi:10.3390/ijms27104599)
Supplement: Supplementary file 1 [file ijms-27-04599-s001.zip › ijms-4260482-supplementary.pdf]

Supplementary Materials

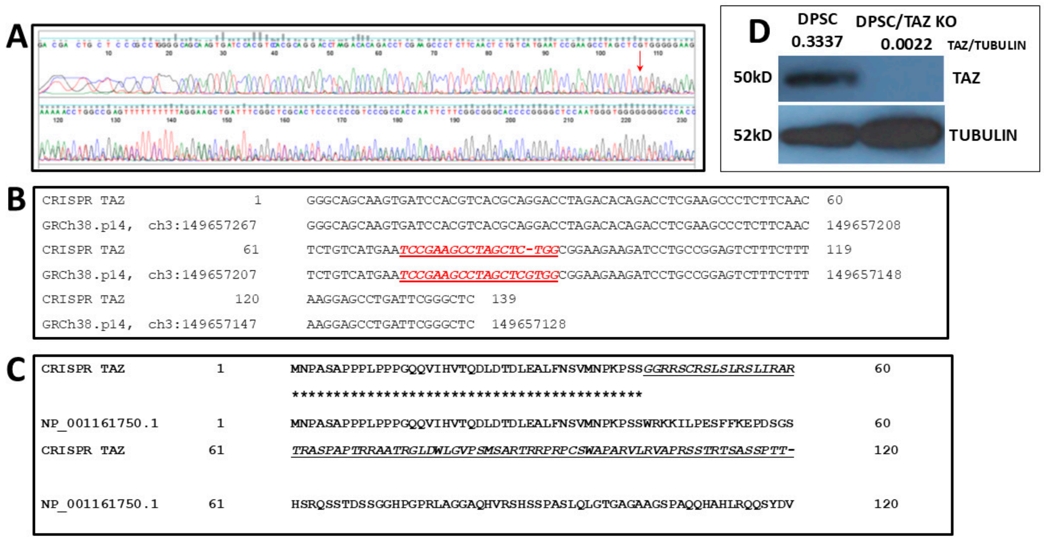

Supplemental Figure S1

Supplemental Figure S1. Generating a *TAZ* gene editing DPSC by CRISPR: A and **B**: DNA Sanger sequencing chromatography of PCR product rendering CRISPR-Cas9 target region and correlated nucleonic acid alignment. Red arrow indicated a deletion (**A**). The target DNA was shown as Red and underlined, and a "g" deletion showed as an underlined hyphen (**B**). **C**: Amino acid alignment of CRISPR TAZ (putative mutant TAZ) and NP_001161750.1 (WT TAZ). Identical AAs were indicated as * up to S_{42}, underlined AAs were putative AAs and a "-" indicated a $STOP_{120}$. **D**: Reduced TAZ expression detected by WB. TUBULIN was used as loading control.

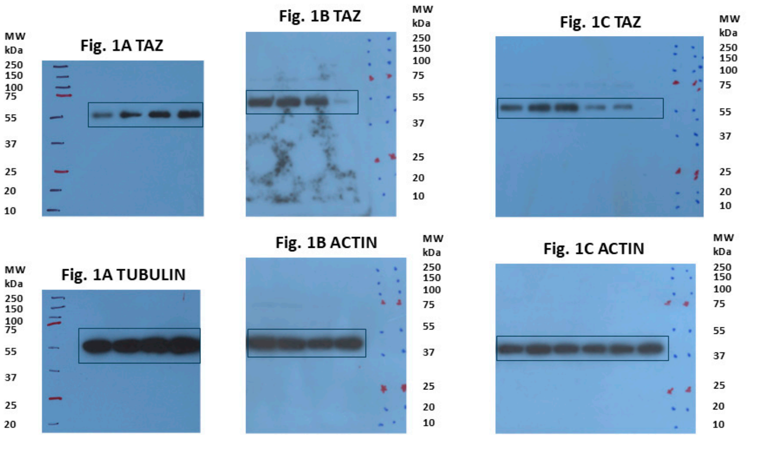

Full length images for Figure 1 with the squares showing the cropped regions

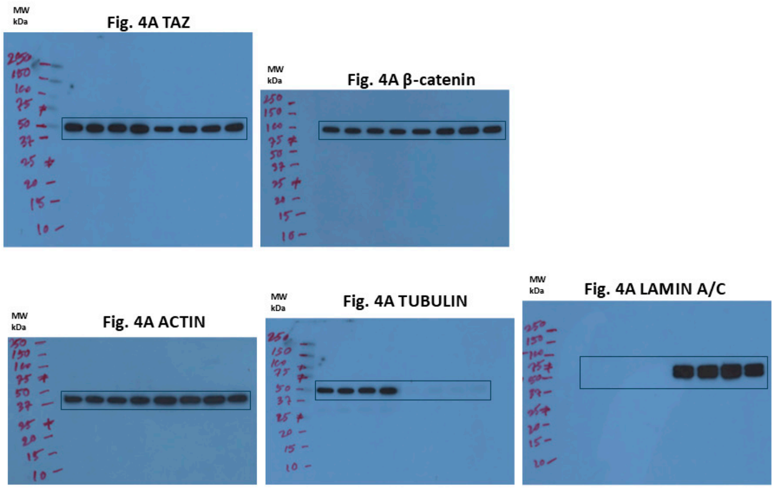

Full length images for Figure 4A with the squares showing the cropped regions

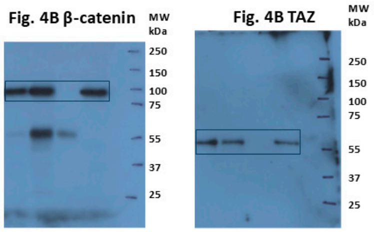

Full length images for Figure 4B with the squares showing the cropped regions

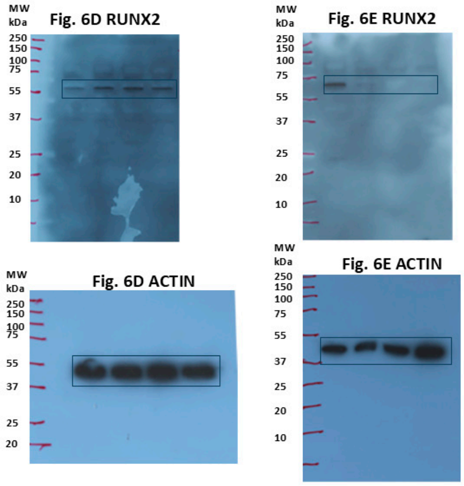

Full length images for Figure 6 with the squares showing the cropped regions

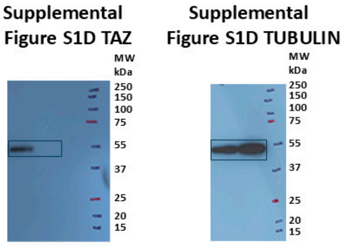

Full length images for Supplemental Figure S1D with the squares showing the cropped regions

49 Spadina Ave. Suite 200
Toronto ON M5V 2J1 Canada
www.biorender.com

Confirmation of Publication and Licensing Rights - Open Access

February 20th, 2026

Subscription Type:	*Student Plan - Academic*
Agreement number:	*QM29DWKAPT*
Publisher Name:	*Scientific Reports*
Figure Title:	*Figure 10. Hypothetical Figure*
Citation to Use:	*Created in BioRender. Villani, C. (2026) https://BioRender.com/1mqp1tb*

To whom this may concern,

This document ("Confirmation") hereby confirms that Science Suite Inc. dba BioRender ("BioRender") has granted the following BioRender user: Cassandra Villani ("User") a BioRender Academic Publication License in accordance with BioRender's Terms of Service and Academic License Terms ("License Terms") to permit such User to do the following on the condition that all requirements in this Confirmation are met:

1) publish their Completed Graphics created in the BioRender Services containing both User Content and BioRender Content (as both are defined in the License Terms) in publications (journals, textbooks, websites, etc.); and
2) sublicense such Completed Graphics under "open access" publication sublicensing models such as CC-BY 4.0 and more restrictive models, so long as the conditions set forth herein are fully met.

Requirements of User:

1) All Completed Graphics to be published in any publication (journals, textbooks, websites, etc.) must be accompanied by the following citation either as a caption, footnote or reference for each figure that includes a Completed Graphic:
   "Created in BioRender. Villani, C. (2026) https://BioRender.com/1mqp1tb ".
2) All terms of the License Terms including all Prohibited Uses are fully complied with. E.g. For Academic License Users, no commercial uses (beyond publication in journals, textbooks or websites) are permitted without obtaining or switching to a BioRender Industry Plan.
3) A Reader (defined below) may request that the User allow their figure to be a public template for Readers to view, copy, and modify the figure. It is up to the User to determine what level of access to grant.

Open-Access Journal Readers:

Open-Access journal readers ("Reader") who wish to view and/or re-use a particular Completed Graphic in an Open-Access journal subject to CC-BY sublicensing may do so by clicking on the URL link in the applicable citation for the subject Completed Graphic.
